# High Affinity Promotes Internalization of Engineered Antibodies Targeting FGFR1

**DOI:** 10.3390/ijms19051435

**Published:** 2018-05-10

**Authors:** Łukasz Opaliński, Jakub Szymczyk, Martyna Szczepara, Marika Kucińska, Daniel Krowarsch, Małgorzata Zakrzewska, Jacek Otlewski

**Affiliations:** 1 Department of Protein Engineering, Faculty of Biotechnology, University of Wroclaw, Joliot-Curie 14a, 50-383 Wroclaw, Poland; jakub.szymczyk@uwr.edu.pl (J.S.); martyna.szczepara@uwr.edu.pl (M.S.); kucinska.marika@gmail.com (M.K.); malgorzata.zakrzewska@uwr.edu.pl (M.Z.); 2 Department of Protein Biotechnology, Faculty of Biotechnology, University of Wroclaw, Joliot-Curie 14a, 50-383 Wroclaw, Poland; daniel.krowarsch@uwr.edu.pl

**Keywords:** affinity, cancer therapy, engineered antibodies, FGFR1, internalization

## Abstract

Fibroblast growth factor receptor 1 (FGFR1) is a plasma membrane protein that transmits signals from the extracellular environment, regulating cell homeostasis and function. Dysregulation of FGFR1 leads to the development of human cancers and noncancerous diseases. Numerous tumors overproduce FGFR1, making this receptor a perspective target for cancer therapies. Antibody-drug conjugates (ADCs) are highly potent and selective anticancer agents. ADCs are composed of antibodies (targeting factors) fused to highly cytotoxic drugs (warheads). The efficiency of ADC strategy largely depends on the internalization of cytotoxic conjugate into cancer cells. Here, we have studied an interplay between affinity of anti-FGFR1 antibodies and efficiency of their cellular uptake. We have developed a unique set of engineered anti-FGFR1 antibodies that bind the same epitope in the extracellular part of FGFR1, but with different affinities. We have demonstrated that these antibodies are effectively taken up by cancer cells in the FGFR1-dependent manner. Interestingly, we have found that efficiency, defined as rate and level of antibody internalization, largely depends on the affinity of engineered antibodies towards FGFR1, as high affinity antibody displays fastest internalization kinetics. Our data may facilitate design of therapeutically relevant targeting molecules for selective treatment of FGFR1 overproducing cancers.

## 1. Introduction

Cancer is one of the top causes of mortality worldwide. Currently, nearly one in six deaths is due to cancer and it is expected that the number of new cases will rise by 70% in the coming two decades [1]. Traditional anti-cancer therapies usually aim for inhibition of high proliferative capacity of cancer cells. However, most of the targeted pathways are also critical for maintenance of normal cells, thus giving rise to numerous side effects of conventional anti-cancer drugs. In recent years, engineered monoclonal antibodies and antibody fragments have attracted attention as molecules that may ensure specificity of the cancer treatment [2]. Such antibodies inactivate the specific receptor on cancer cells, resulting in induction of apoptosis, or lead to cancer cell death by stimulation of the immune system of the patient [2]. Alternatively, engineered antibodies may be physically linked to highly potent cytotoxic drugs in the antibody-drug conjugates (ADCs). In the ADC approach, antigen-positive cancer cells are recognized by the antibody part of the ADCs. Next, ADCs bound to the cell surface antigen are internalized, utilizing one of cellular endocytic routes. Subsequently, ADCs traffic via cellular vesicular compartments to their final lysosomal destination, where proteolytic degradation releases cytotoxic drugs from the ADCs. Drug moiety diffuses out from lysosomes and binds its intracellular target causing cell death [3,4]. Therefore, the effectiveness of ADC therapy depends on the selectivity and strength of antigen binding, tumor penetration and on the efficiency of ADCs internalization from the cell surface [5,6,7,8,9].

The fibroblast growth factor receptors comprise a group of four conserved receptor tyrosine kinases (FGFR1-FGFR4) that, in conjunction with extracellular fibroblast growth factors (FGFs), transmit signals across the plasma membrane. Binding of FGFs to FGFRs (fibroblast growth factor receptors) leads to the activation of the receptor cytoplasmic tyrosine kinase domain that recruits numerous signaling molecules further propagating the signal [10,11]. The FGFR-dependent signaling cascades govern cell metabolism, proliferation, and apoptosis and are critical for angiogenesis, organogenesis, and wound healing [12]. The aberrations in the FGFRs such as gene amplification, rearrangements, and somatic mutations are often observed in cancer and can be found in over 7% of all tumors [13].

FGFR1 is an attractive target for selective chemotherapy in ADC, as it is localized on the cell surface, thus being easily accessible to extracellular targeting molecules [10,11]. In numerous cancer cell types, the level of FGFR1 is elevated in comparison to the normal cells that may ensure selectivity of drug targeting [14]. Moreover, FGFR1 is rapidly internalized, mainly via clathrin-mediated endocytosis, providing the intracellular release of the drug after lysosomal degradation of ADC inside cancer cells [15]. The requirements for the design of highly internalizing antibodies against FGFR1 suitable as an ADC’s carrier are still largely undefined. We have recently developed novel antibody fragment scFvD2-Fc and have demonstrated that bivalency of scFvD2-Fc promotes internalization of this anti-FGFR1 engineered antibody by inducing receptor dimerization [16,17]. Here, we have assessed the importance of engineered antibodies affinities towards FGFR1 for their internalization. Using the phage display approach, we have selected two novel FGFR1-specific antibodies that bind to the same epitope within extracellular part of FGFR1 as scFvD2-Fc, but with different affinities. We have demonstrated that all these engineered antibodies are efficiently internalized via receptor-mediated endocytosis and are delivered through endosomes to lysosomes. Interestingly, our data show that an antibody with the highest affinity to FGFR1 displays the fastest internalization rate. Taken together, our data may facilitate the effective design of highly internalizing engineered antibodies suitable for ADC strategy of cancer treatment.

## 2. Results

### 2.1. Engineered Antibody Fragments Recognize the Same Epitope within D1 Domain of the FGFR1

To select the panel of antibody fragments that specifically recognize FGFR1, we employed the phage display technique using Tomlinson I and Tomlinson J libraries and the extracellular part of FGFR1 (composed of D1, D2, and D3 domains) fused to the Fc fragment (FGFR1.D1-D2-D2-Fc) as an antigen [18]. For each library, we performed three consecutive rounds of selection. We obtained four novel scFv proteins that interact with the extracellular region of FGFR1: scFvK10, scFvL8, scFvL12 and scFvP4.

**Figure 1:**
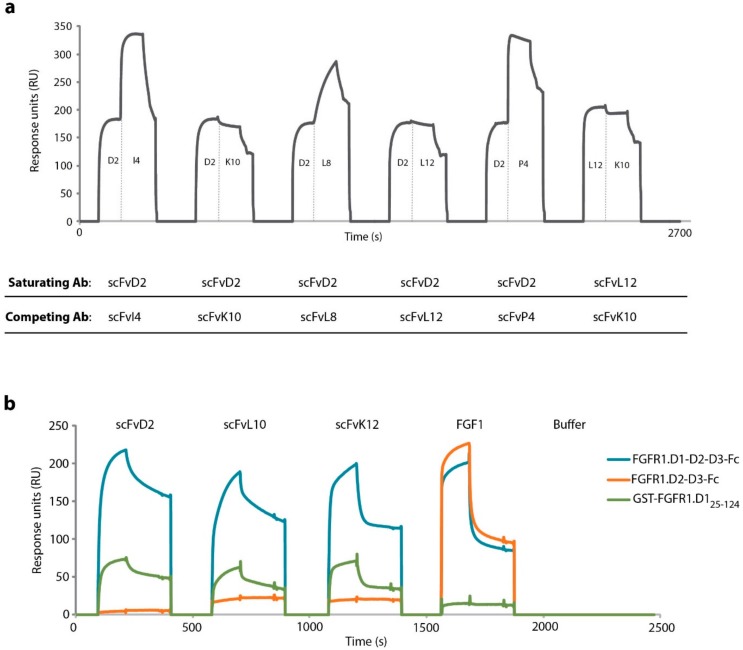

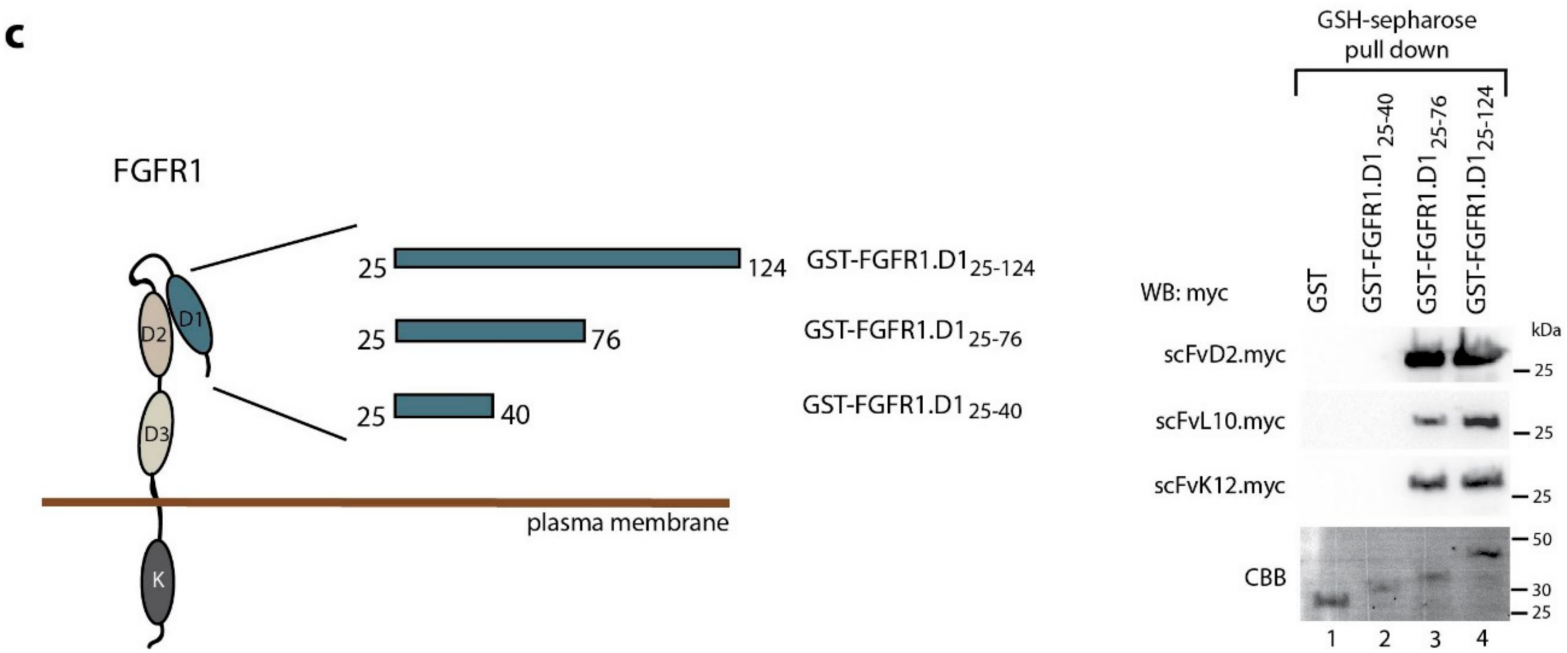
Engineered antibodies recognize the same epitope within the extracellular region of FGFR1. (**a**) SPR-based epitope binning. FGFR1.D1-D2-D3-Fc was immobilized on sensors and incubated with high concentrations of saturating antibodies. Next, competing antibodies were injected to assess if their binding to the extracellular region of FGFR1 is blocked by saturating antibodies. (**b**) SPR analysis of the interaction of scFv proteins with FGFR1 truncations. CM5 sensors were coated with FGFR1.D1-D2-D3-Fc, FGFR1.D2-D3-Fc, or GST-FGFR1-D1_25–124_, and the association and dissociation were monitored for 240 s after injection of scFv proteins (1 µM), or FGF1 (1 µM). (**c**) Pull down experiments with scFv proteins and truncated variants of the D1 domain of FGFR1. GST and GST-tagged truncated versions of the D1 domain were bound to Glutathione Sepharose and incubated with scFv proteins. After extensive washing, bound proteins were eluted and analyzed by Western blotting. Membranes were first stained with CBB to visualize eluted proteins and then detected with anti-c-myc antibodies (for visualization of scFv proteins containing C-teminal c-myc).

Our group has recently selected and characterized scFvD2 as a high affinity FGFR1 interactor [16]. To obtain panel of scFv fragments that bind to the same region of the extracellular part of FGFR1 as scFvD2 we employed epitope binning using surface plasmon resonance (SPR). We intended to select scFv proteins whose interaction with FGFR1-D1-D2-D3-Fc was blocked by the saturating concentrations of scFvD2. To this end, sensors with immobilized FGFR1-D1-D2-D3-Fc were incubated first with high concentration (1 µM) of scFvD2 and then with novel anti-FGFR1 scFv proteins at the same concentration. We observed that binding of scFvD2 to the extracellular part of FGFR1 inhibited receptor interaction with scFvK10 and scFvL12 (Figure 1a). Moreover, the saturation of FGFR1-D1-D2-D3-Fc with scFvL12 blocked subsequent binding of scFvK10, suggesting that these three antibody fragments compete for the same binding site on FGFR1 (Figure 1a).To identify the epitope recognized by these three scFv proteins, we performed SPR binding studies with sensor-immobilized full length extracellular part of FGFR1 (FGFR1.D1-D2-D3-Fc) and truncated receptor variant lacking D1 domain (FGFR1.D2-D3-Fc). The lack of the D1 domain abolished the interaction of scFvD2, scFvK10, and scFvL12 with immobilized FGFR1 fragment, indicating that these proteins recognize epitope within the D1 domain of the receptor (Figure 1b). We confirmed this finding, showing that all three engineered antibodies bind to sensor-immobilized purified D1 domain fused to GST (GST-FGFR1.D1_25–124_) (Figure 1b).

To validate our experiments, we used FGF1, a natural FGFR1 ligand that recognizes binding site formed by D2 and D3 domains of the receptor. As expected, FGF1 interacted with FGFR1.D1-D2-D3-Fc and FGFR1.D2-D3-Fc, but did not bind to GST-FGFR1.D1_25–124_ (Figure 1b). To identify the precise epitope within the D1 domain recognized by scFv proteins, we prepared a set of C-terminal truncations of D1 domain fused to glutathione S-transferase GST (Figure 1c). Next, we performed pull down experiments with purified GST-tagged D1 domain truncations and GST as a control. We found that scFvD2, scFvK10, and scFvL12 bound to the full length D1 domain (GST-FGFR1.D1_25–124_) and to the D1 truncation composed of residues 25–76 (GST-FGFR1.D1_25–76_). The interaction was not observed when only N-terminal peptide, containing residues 25–40, was applied (GST-FGFR1.D1_25–40_) (Figure 1c). These data demonstrate that engineered anti-FGFR1 antibodies recognize epitope that is located within residues 41–76 of the D1 domain of FGFR1. 

**Figure 2:**
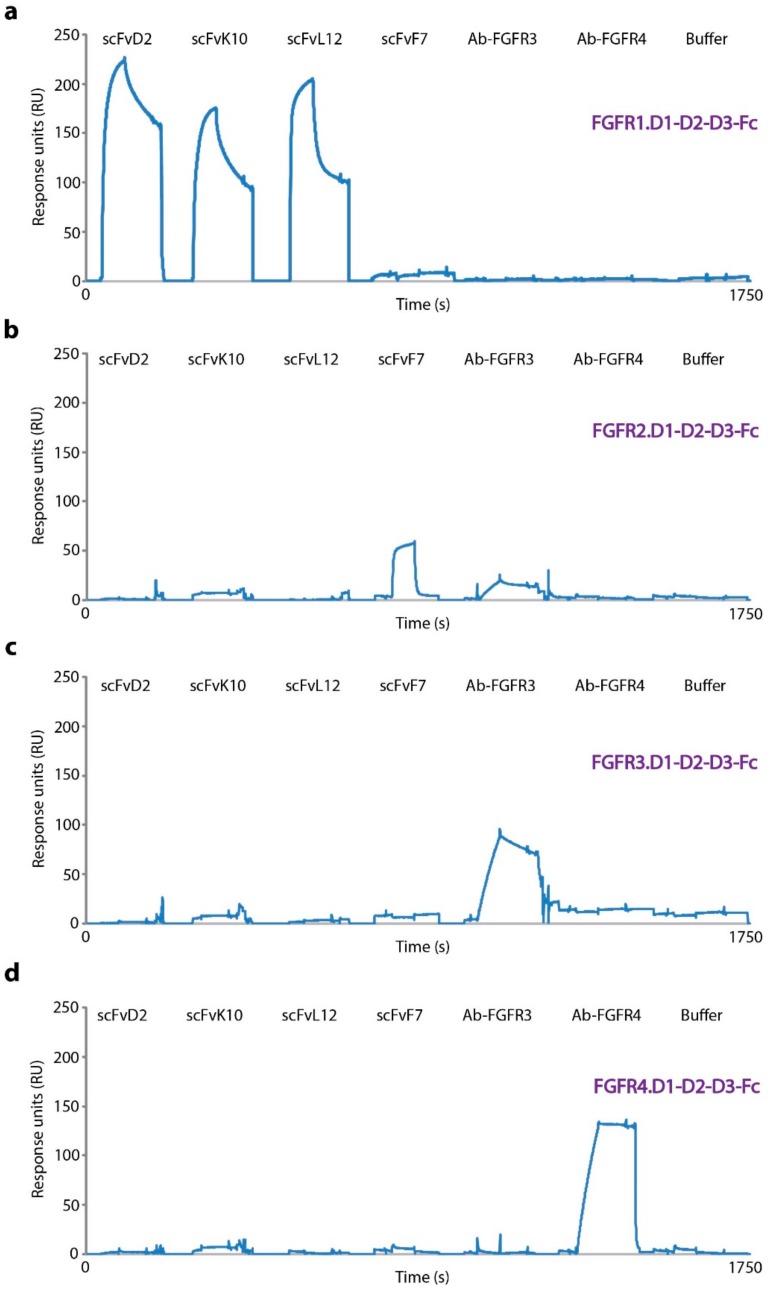
Specificity of scFv proteins towards FGFR1. Extracellular regions of FGFR1 (FGFR1.D1-D2-D3-Fc (**a**); FGFR2 (FGFR2.D1-D2-D3-Fc (**b**); FGFR3 (FGFR3.D1-D2-D3-Fc (**c**); and FGFR4 (FGFR4.D1-D2-D3-Fc (**d**); were immobilized on CM5 sensors and incubated with scFvD2, scFvL10, scFvK12, scFvF7 (specific towards FGFR2), and commercial anti-FGFR3 and anti-FGFR4 antibodies.

Next, we assessed specificity of scFvD2, scFvK10, and scFvL12 towards FGFR1 using SPR. Using extracellular regions of all four FGF receptors (FGFR1-4) immobilized on CM5 sensors, we observed binding of scFvD2, scFvK10, and scFvL12 only to FGFR1 (Figure 2). The high selectivity of scFv-Fc proteins towards FGFR1 can be attributed to the very low homology of FGF receptors within residues 41–76 (13.9% identity, while sequence identity in the full extracellular region of FGF receptor is 36.7%) (Figure S1). To confirm that immobilized fragments of FGF receptors were functional, we employed developed in our group scFvF7 that recognizes FGFR2 [19] as well as commercial anti-FGFR3 and anti-FGFR4 antibodies as controls (Figure 2 and Figure S2).

These results demonstrate that three independently selected scFv proteins display high specificity towards FGFR1 and recognize the same epitope (residues 40–76) within the D1 domain of FGFR1.

### 2.2. Engineered Antibodies Differ in the Affinity Towards FGFR1

We have recently demonstrated that reformatting of the scFvD2 into bivalent scFvD2-Fc largely improved internalization of this engineered antibody by inducing FGFR1 dimerization [16,17]. To study the impact of antibody affinity to FGFR1 on the internalization of antibody-receptor complexes, we have also prepared Fc-fusions of scFvK10 and scFvL12 (scFvK10-Fc and scFvL14-Fc). Proteins were expressed in chinese hamster ovary (CHO) cells and purified using affinity chromatography. Next, the binding of scFvK10-Fc and scFvL12-Fc to the extracellular region of FGFR1 (FGFR1.D1-D2-D3-Fc) were analyzed by SPR technique. The calculated kinetic parameters (k_on_, k_off_ and K_D_) clearly demonstrate that scFvD2-Fc displays the highest affinity towards FGFR1 (0.59 nM) [16], while scFvK10-Fc and scFvL12-Fc bind to the FGFR1 with over tenfold weaker affinities than scFvD2-Fc (9.41 nM and 13.4 nM, respectively) (Table 1, Figure S3).

### 2.3. Antibody Fragments Are Internalized via Receptor Mediated Endocytosis

**Figure 3:**
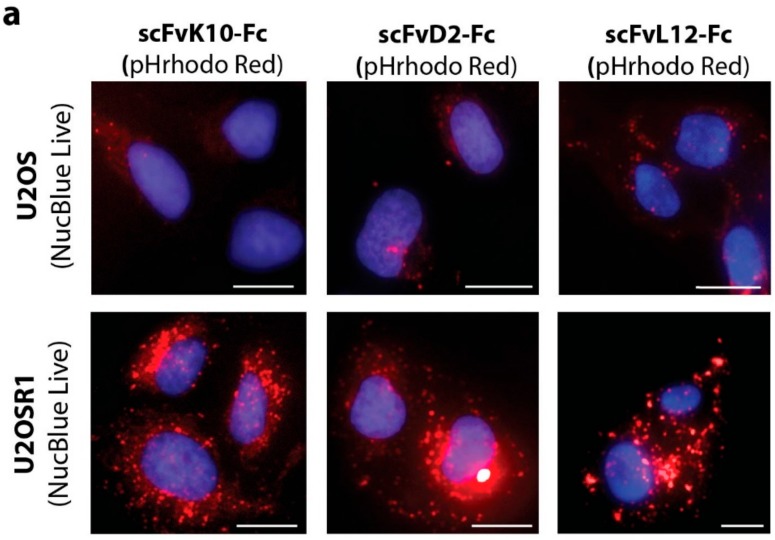

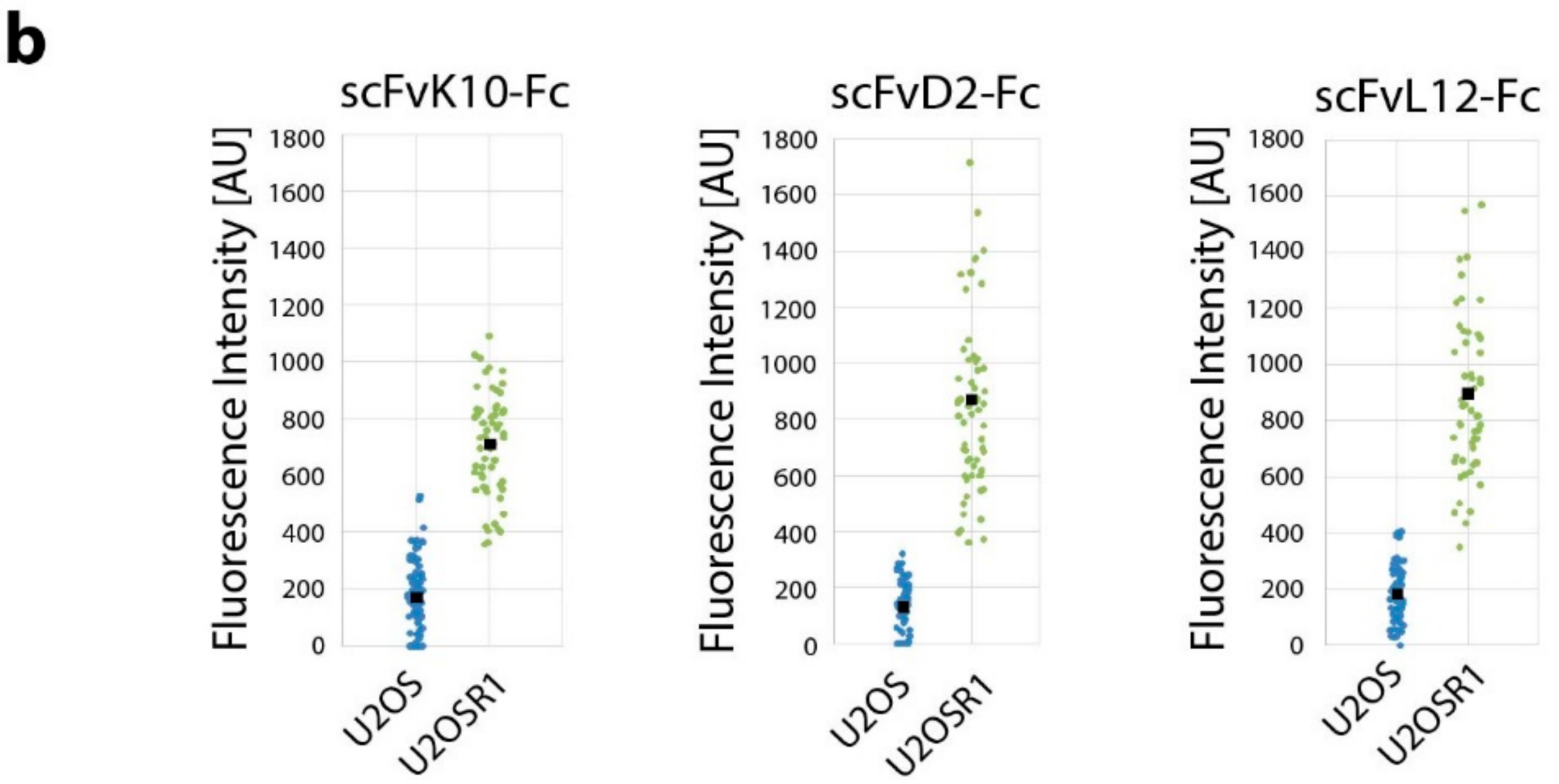
FGFR1-dependence of scFv proteins internalization. (**a**) U2OS (control) and U2OSR1 cells (overproducing FGFR1) were incubated for 2 h with pHrodo Red labeled scFv proteins (red). Nuclei were stained with NucBlue Live (blue) and cells were analyzed by fluorescence microscopy. Scale bars represent 20 µm. (**b**) Quantification of internalization of pHrodo Red-labeled scFvs into U2OS and U2OSR1 cells. pHrodo Red fluorescence intensity inside single cells was measured using Zen 2.3 software (Zeiss, Oberkochen, Germany). At least 30 cells were measured from five fields of view for U2OS and U2OSR1 cells. Each square on the graph represents fluorescence intensity of single cell. Black squares represent average fluorescence intensity for particular cell line.

Next, we studied the internalization of engineered antibody fragments. For this purpose, we labeled scFvD2-Fc, scFvK10-Fc, and scFvL12-Fc with pH sensitive fluorophore pHrodo Red. The fluorescence intensity of pHrodo Red is very low at neutral or basic pH (outside the cells or on the cell surface), but largely increases in the acidic milieu of the endosomal and lysosomal lumen (when labeled antibody is internalized) [20]. Fluorescently labeled engineered antibodies were incubated with model U2OSR1 cells (overproducing FGFR1) and control U2OS cells (with very low level of FGF receptors) and analyzed by fluorescence microscopy. Internalization of scFv-Fc proteins was minimal for U2OS cells, while all three studied engineered antibodies were efficiently taken up by U2OSR1 cells (Figure 3a). Quantitative analysis of fluorescence intensities confirmed that all three scFv-Fc proteins required FGFR1 for their internalization (Figure 3b).

**Figure 4:**
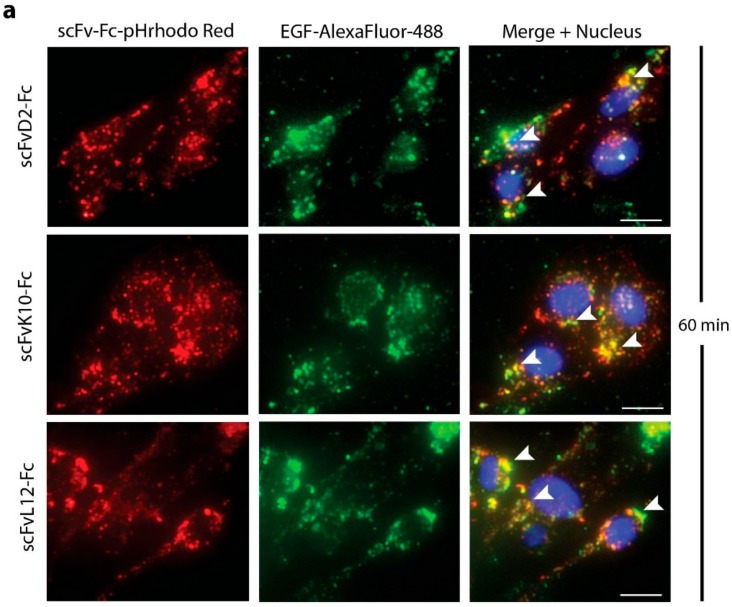

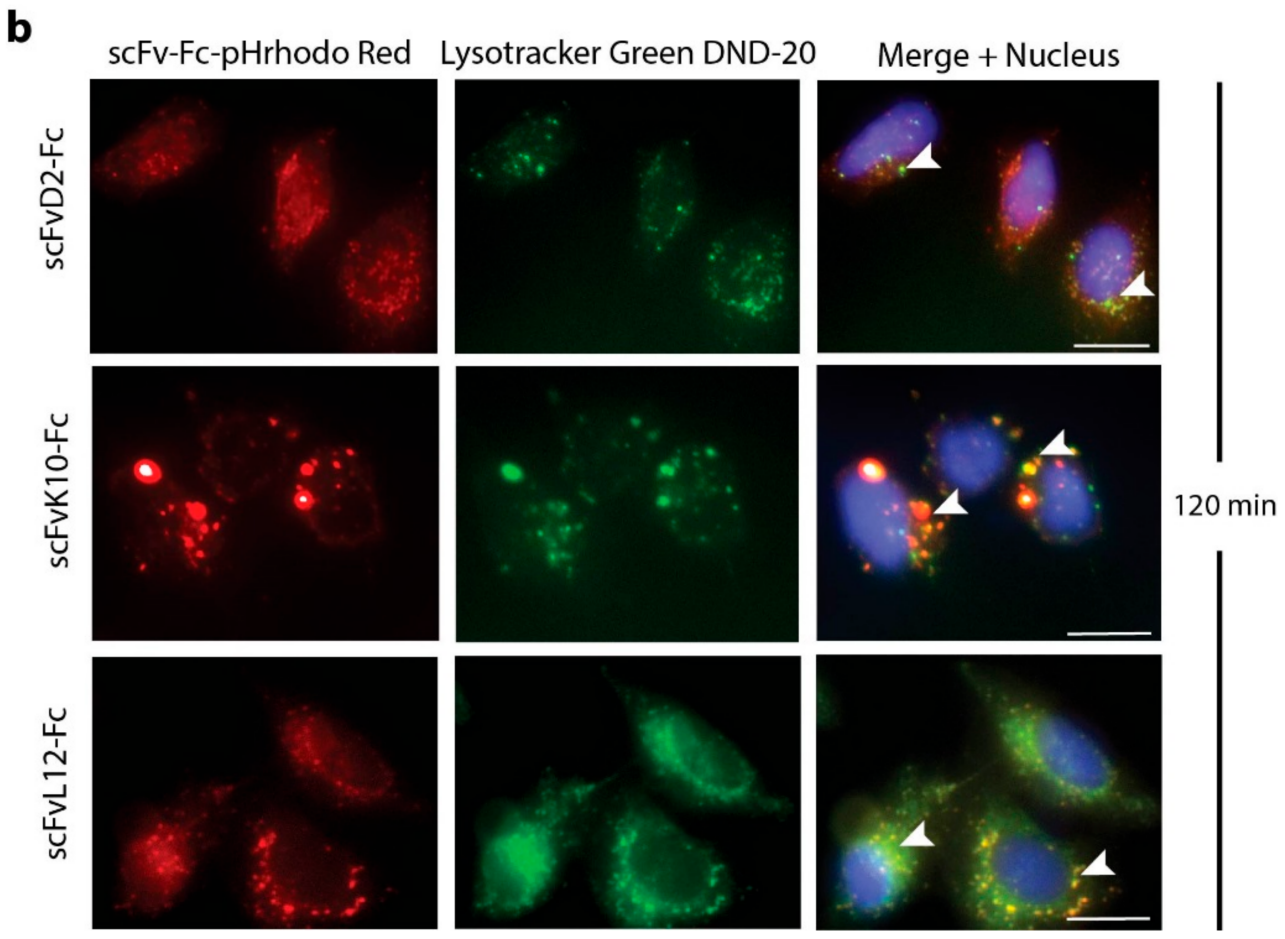
Intracellular trafficking of engineered antibodies. (**a**) U2OSR1 cells were incubated for 1 h with pHrodo Red labeled scFv proteins (red) and EGF-AlexaFluor-488 (green) (marker of internalization pathway directing cargo for lysosomal degradation). Nuclei were stained with NucBlue Live (blue) and cells were analyzed by fluorescence microscopy. Scale bars represent 20 µm. (**b**) U2OSR1 cells were incubated for 2 h with pHrodo Red labeled scFv proteins (red). Lysosomes were subsequently stained with Lysotracker Green DND-20 (green) and nuclei were labeled with NucBlue Live (blue). Cells were analyzed by fluorescence microscopy. Arrowheads mark selected regions of high degree of signal colocalization. Scale bars represent 20 µm.

Upon internalization, engineered antibodies can be directed to recycling endosomes, or to endosomes that fuse with lysosomes for degradation [15]. As epidermal growth factor (EGF) is a marker of endosomes following the degradation pathway, we simultaneously applied fluorescently labeled EGF (EGF-AlexaFluor-488) to visualize these compartments [21]. We observed a high degree of colocalization of pHrodo Red-labeled scFvD2-Fc, scFvK10-Fc, and scFvL12-Fc with EGF-AlexaFluor-488, which suggests that engineered antibodies are sequestered into endosomal compartments destined for fusion with lysosomes (Figure 4a). As expected, at longer time points (120 min) we observed colocalization of pHrodo Red-labeled antibody fragments with the lysosome-specific dye–Lysotracker Green DND-20 (Figure 4b). All these data demonstrate that engineered antibodies are internalized via FGF receptor-mediated endocytosis and are directed to lysosomes for degradation.

### 2.4. High Affinity Promotes Internalization of Engineered Antibodies

**Figure 5:**
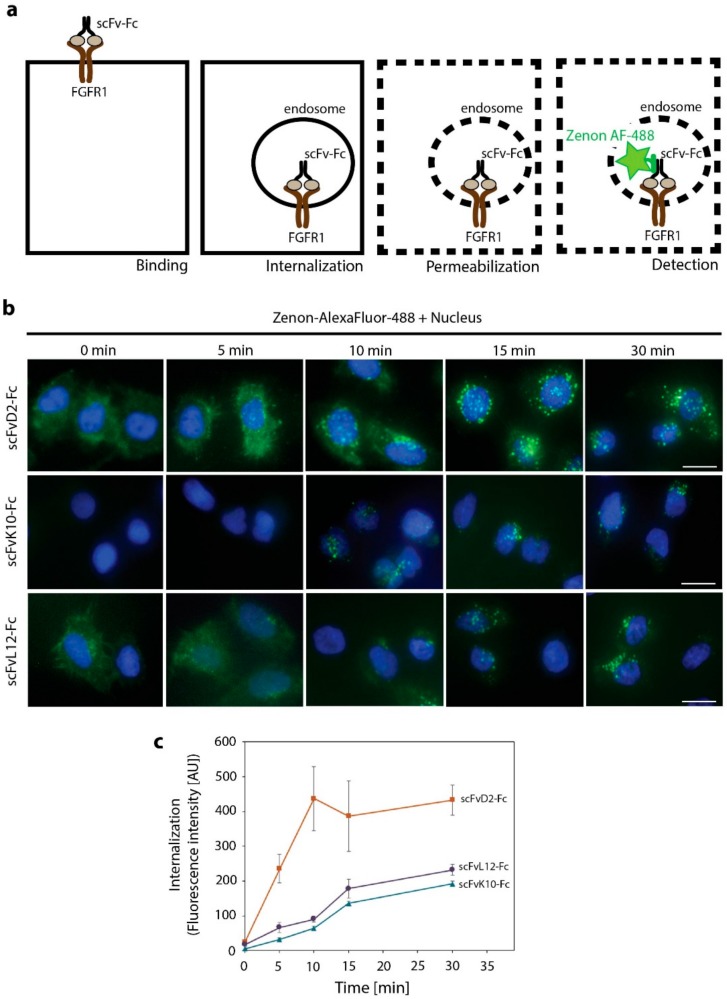
Kinetics of engineered antibodies internalization. (**a**) Scheme of the experimental setup. Non-labeled scFv-Fc proteins were incubated with U2OSR1 cells to allow for binding and internalization. Next, cell surface-bound engineered antibodies were removed by washing, cells were permeabilized and incubated with Zenon-AlexaFluor-488, a fluorescently labeled Fab fragment specifically recognizing Fc fragment of human origin that detects non-labeled, internalized scFv-Fc proteins. (**b**) U2OSR1 cells were incubated with scFv-Fc proteins for various time points and internalized engineered antibodies were detected with Zenon-AlexaFluor-488 (green), as described above. Nuclei were labeled with NucBlue Live (blue). Scale bars represent 20 µm. (**c**) Kinetics of scFv-Fc proteins internalization. Fluorescence intensity of Zenon-AlexaFluor-488 (visualizing internalized scFv-Fc proteins) was measured within single U2OSR1 cells at various time points. At least 20 cells were measured from three fields of view for each time point. The values represent average fluorescence intensity from three experiments per cell. Error bars represent +/− SEM.

As differences in the efficiency of chemical labeling made the quantitative comparison of the internalization rates of fluorescently labeled antibodies impossible, we developed a fluorescence microscopy assay where we utilized non-labeled scFv-Fc proteins. Engineered antibodies were incubated with the U2OSR1 cells and the internalization was blocked at various time points by placing the cells on ice. Surface-bound antibodies were subsequently removed by washing the cells with buffer containing high salt concentration and low pH (HSLP). Cells were then fixed, permeabilized, and incubated with Zenon-AF-488, fluorescently labeled Fab fragment specifically recognizing the Fc fragment of the human origin, to visualize internalized scFv-Fc proteins (Figure 5a).

We observed that scFvD2-Fc displayed the fastest kinetics of internalization and the highest accumulation within U2OSR1 cells (Figure 5b,c). The internalization of the other two engineered antibodies (scFvK10-Fc and scFvL12-Fc) that bind to the same antigen within FGFR1, but with about 15 times lower affinity, was much slower and lower amounts of antibodies were taken up by the cells (Figure 5b,c). These data demonstrate that efficiency of the receptor-mediated endocytosis of anti-FGFR1 antibodies largely depends on the affinity of antibodies towards the receptor.

## 3. Discussion

Success in selective targeting of tumors with engineered antibodies and their conjugates largely depends on the properties of the cancer antigen and applied antibody. The characteristics of an “ideal” antigen for ADC strategy are still under the debate. Nevertheless, the “ideal” antigen is believed to be present at an elevated level on the surface of cancer cells, efficiently endocytosed and targeted to lysosomes [3,4,5]. On the other hand, the perfect targeting molecule (antibody), especially in the ADC approach, should display high affinity towards specific cancer marker (ensuring selectivity) and high internalization rate, facilitating the efficient delivery of its cargo (cytotoxic drug) [3,4,5]. The relationship between affinity of antibodies to cell surface antigens and their internalization kinetics is still not established. It was demonstrated that high affinity to antigen promotes internalization of antibodies directed against CD44 and HER2 receptors [22,23]. In contrast, differences in binding to target had no influence of the internalization rates of anti-CEA scFv proteins [24]. Moreover, the significance of antibodies affinities for tumor targeting in vivo is controversial. Some high affinity antibodies display poor tumor penetration, while in the other cases high affinity supports tumor targeting by antibodies [6,7,8,9]. Therefore, in case of each antigen–antibody pair it is important to assess the antigen properties and the significance of interaction strength for the efficiency of antibody internalization and for tumor targeting in vivo.

Here, we have analyzed the interplay between the affinity of anti-FGFR1 antibody fragments to the receptor and their cellular uptake. We have developed a unique set of engineered antibodies that bind to the same epitope of extracellular part of FGFR1, but with different strength. All these antibodies are internalized by FGFR1-dependent endocytosis, however high affinity antibody is taken up by the cells more rapidly than antibodies displaying lower affinities towards FGFR1. We have recently demonstrated that bivalent anti-FGFR1 antibodies are rapidly internalized by promoting receptor dimerization that constitutes the trigger for receptor endocytosis [17]. In our experiments, we used excessive concentrations of scFv-Fc proteins (over 20 times higher than equilibrium dissociation constants), thus a vast majority of the cell surface FGFR1 was occupied by antibody fragments. Therefore, we hypothesize that rapid internalization of the high affinity bivalent antibody-FGFR1 complexes is caused by enhanced FGFR1 dimerization on the cell surface.

Taken together, our data imply that bivalency and high affinity ensure efficient internalization of engineered anti-FGFR1 antibodies. These results may facilitate design of targeting molecules suitable for ADCs directed against FGFR1-overproducing cancers.

## 4. Materials and Methods

### 4.1. Antibodies and Recombinant Proteins

The primary anti-c-myc antibodies (sc-40), anti-FGFR3 (sc-73994), and anti-FGFR4 (sc-73995) were from Santa Cruz Biotechnology (Dallas, TX, USA). The primary antibody against FGFR1 was generated by Davids Biotechnologie GmbH (Regensburg, Germany) [17]. Recombinant FGF1 (Met-Ala-FGF122-155) was produced in *E. coli*, as described previously [25]. The Fc fragment and the full length extracellular region of FGFR1 and the extracellular part of FGFR1 lacking N-terminal D1 domain fused to the Fc were produced in CHO cells and purified using Protein A Sepharose (GE Healthcare, Piscataway, NJ, USA) [18]. 

Antibody fragments in the scFv format (scFvD2, scFvI4, scFvK10, scFvL8, scFvL12, scFvP4, scFvF7 containing c-myc) were expressed in *E. coli* HB2151 and purified using Protein A Sepharose. scFv proteins in the bivalent Fc format (scFvD2-Fc, scFvK10-Fc and scFvL12-Fc) were produced in CHO cells and purified with Protein A Sepharose [16]. 

The DNA sequence encoding the D1 domain of FGFR1 (residues 25–124) was cloned into pDEST15 using Gateway Cloning (Thermo Fisher Scientific; Waltham, MA, USA), resulting in plasmid pEXP15-D1_25–124_ (allowing for production of GST-FGFR1-D1_25–124_). Plasmids for production of truncated variants of D1 domain: pEXP-D1_25–76_ (for production of GST-FGFR1-D1_25–76_) and pEXP-D1_25–40_ (for production of GST-FGFR1-D1_25–40_) were generated by introducing stop codon in the vector pEXP15-D1_25–124_ by site directed mutagenesis. GST-tagged truncated variants of the D1 domain were produced in *E. coli* BL21 CodonPlus(DE3)-RIL (Agilent Technologies; Santa Clara, CA, USA) and purified with Glutathione Sepharose (GE Healthcare, Piscataway, NJ, USA), according to the standard procedure.

### 4.2. Cells

U2OS (human osteosarcoma, ATCC #HTB-96) and U2OSR1 cells (U2OS cells stably expressing FGFR1) were a kind gift of Dr. E.M. Haugsten from the Department of Molecular Cell Biology, Institute for Cancer Research, Oslo University Hospital, Norway. Cells were cultured in 5% CO_2_ atmosphere at 37 °C in Dulbecco’s Modified Eagle’s Medium (Biowest, Nuaille, France) supplemented with 10% fetal bovine serum (Thermo Fisher Scientific, Waltham, MA, USA), antibiotics mix (100 U/mL penicillin, 100 µg/mL streptomycin (Thermo Fisher Scientific; Waltham, MA, USA), and 1 mg/mL geneticin (in the case of U2OSR1 cells) (Thermo Fisher Scientific; Waltham, MA, USA). Cells were seeded into tissue culture plates one day prior start of the experiments.

### 4.3. Selection of Antibody Fragments against FGFR1 by Phage Display Technology

The selection of scFv proteins recognizing an extracellular region of FGFR1 was carried out using two commercial phage libraries of human scFv (Tomlinson I and J) as described previously [16]. Three cycles of panning were performed on MaxiSorp 96-well plates (Thermo Fisher Scientific; Waltham, MA, USA) coated with FGFR1.D1-D2-D3-Fc with counter selection using purified Fc protein. To confirm the interaction with FGFR1 bacterial supernatants containing selected scFv proteins were subjected to the monoclonal ELISA and the SPR assay with immobilized FGFR1.D1-D2-D3-Fc, as described in [16].

### 4.4. SPR-Based Interaction Studies and Affinity Measurements

SPR experiments were performed on the Biacore 3000 instrument (GE Healthcare, Piscataway, NJ, USA) at 25 °C. For epitope binning FGFR1.D1-D2-D3-Fc was immobilized on a CM5 sensor (GE Healthcre) at 825 RU and scFv proteins (1 µM) were sequentially injected at flow rate of 30 µL/min. The association and dissociation were monitored for 240 s.

For analysis of the binding site of scFv proteins within FGFR1, CM5 sensors were coated with FGFR1.D1-D2-D3-Fc (at 825 RU), FGFR1.D2-D3-Fc (at 900 RU), or GST-FGFR1-D1_25–124_ (at 300 RU). Next, scFv proteins (1 µM) and FGF1 (1 µM) were injected and the association and dissociation were monitored for 240 s.

For specificity analysis of generated scFv proteins, FGFR1.D1-D2-D3-Fc (at 825 RU), FGFR2.D1-D2-D3-Fc (at 1000 RU), FGFR3.D1-D2-D3-Fc (at 1000 RU), and FGFR4.D1-D2-D3-Fc (at 1000 RU) were immobilized on CM5 sensors. Next, scFv proteins (1 µM), anti-FGFR1 antibody (as a positive control for interaction with FGFR1.D1-D2-D3-Fc) [17], scFvF7 (1 µM) (as a positive control for interaction with FGFR2.D1-D2-D3-Fc) [15] and two commercial antibodies against FGFR3 and FGFR4 (0.1 µM; as positive controls for interaction with FGFR3.D1-D2-D3-Fc and FGFR4.D1-D2-D3-Fc, respectively) were injected independently over all sensors at 30 µL/min flow rate. The association and dissociation were monitored for 240 s. All sensograms were analyzed using BIAevaluation 4.1 software (GE Healthcare, Piscataway, NJ, USA).

To determine kinetic parameters of engineered antibodies–FGFR1 interaction, FGFR1.D1-D2-D3-Fc was immobilized at 825 RU on a CM5 sensor. Various concentrations of scFv-Fc proteins (2.5–320 nM) were measured for 300 s at 30 µL/min flow rate. Kinetic constants (ka, kd, and K_D_) were calculated using BIAevaluation 4.1 software using 1:1 Langmuir binding model with drifting baseline.

### 4.5. Pull-Down

To study the interaction of scFv proteins with purified GST-tagged full length D1 domain of FGFR1 and the D1 domain truncations, purified GST (control, 10 µg) and GST-tagged D1 domain variants (10 µg) were diluted in washing buffer (WB): 50 mM Tris, 150 mM NaCl, pH 7.5, and bound to Glutathione Sepharose. Next, scFv proteins (10 µg) in WB were incubated with resin-bound scFv proteins. Resins were extensively washed with WB and bound proteins were eluted with elution buffer: 50 mM Tris, 150 mM NaCl, 20 mM glutathione, pH 7.5. Eluates were analyzed by Western blotting. Before detection membranes were stained with CBB to visualize the amount of eluted GST-tagged truncated variants of the D1 domain and GST. After destaining, membranes were probed with anti-c-myc antibodies.

### 4.6. Fluorescence Microscopy

Purified scFvD2-Fc, scFvK10-Fc, and scFvD12-Fc were chemically labeled with pHrodo Red (Thermo Fisher Scientific; Waltham, MA, USA) according to manufacturer’s protocol. For analysis of the FGFR1-dependence engineered antibodies internalization, U2OS and U2OSR1 cells were incubated with pHrodo Red-labeled engineered antibodies (10 µg/mL) for 2 h at 37 °C. Cells were extensively washed with PBS, fixed with 4% PFA, nuclei were stained with NucBlue Live (Thermo Fisher Scientific; Waltham, MA, USA) and cells were analyzed by fluorescence microscopy. For quantitation of engineered antibodies internalization, pHrodo Red fluorescence intensity was measured within single cells using ZEN 2.3 software (Zeiss, Oberkochen, Germany). At least 30 cells were measured from five fields of view for U2OS and U2OSR1 cells. 

For analysis of the intracellular trafficking of engineered antibodies, serum-starved U2OSR1 cells were incubated for 1 h at 37 °C with pHrodo Red-labeled scFv-Fc proteins (10 µg/mL) and EGF-AlexaFluor-488 (15 µg/mL) (Thermo Fisher Scientific; Waltham, MA, USA). Alternatively, U2OSR1 cells were incubated for 2 h with pHrodo Red labeled scFv-Fc proteins (10 µg/mL) and stained with lysosome-specific dye Lysotracker Green DND-20 (Thermo Fisher Scientific; Waltham, MA, USA) according to manufacturer’s protocol. Nuclei were stained with NucBlue Live and cells were analyzed by fluorescence microscopy.

For analysis of the kinetics of engineered antibodies internalization, non-labeled scFv-Fc proteins and U2OSR1 cells were used. Cells were incubated with scFv-Fc proteins (15 µg/mL) at 37 °C and internalization was stopped at various time points by placing the cells on ice. Cells were then washed extensively with PBS and with HSLP buffer (high salt low pH) (2 M NaCl, 40 mM NaAc, pH 4.0) to remove cell surface-bound antibodies. Next, cells were fixed with 4% PFA and permeabilized with 0.1% Triton X-100 in PBS. Cells were subsequently blocked with 2% BSA in PBS and internalized scFv-Fc proteins were visualized by addition of Zenon-AlexaFluor-488 (Thermo Fisher Scientific; Waltham, MA, USA) that recognizes the Fc fragment of human origin, accordingly to supplier’s protocol. The excessive Zenon-AlexaFluor-488 was blocked with Blocking Reagent and after extensive washing cells were fixed again to preserve scFv-Fc- Zenon-AlexaFluor-488 complexes. Next, nuclei were stained with NucBlue Live and cells were analyzed with fluorescence microscopy. Zenon-AlexaFluor-488 fluorescence intensity within single U2OSR1 cells was measured using ZEN 2.3 software. At least 20 cells were measured from three fields of view for three experiments. Average intensity +/− SEM were plotted.

Wide-field fluorescence microscopy was carried out using a Zeiss Axio Observer Z1 fluorescence microscope (Zeiss, Oberkochen, Germany). Images were taken using LD-Plan-Neofluar 40×/0.6 Korr M27 objective and Axiocam 503 camera. pHrodo Red signal was visualized with a 540/552 nm bandpass excitation filter and a 575/640 nm bandpass emission filter, EGF-AlexaFluor-488, Lysotracker Green DND-20 and Zenon-AlexaFluor-488 signal was visualized with a 450/490 nm bandpass excitation filter and a 550/590 nm bandpass emission filter. NucBlue Live signal was visualized with a 335/383-nm bandpass excitation filter and a 420/470-nm emission filter. Image analysis was carried out using ZEN 2.3 (Zeiss, Oberkochen, Germany) and ImageJ (NIH, Bethesda, USA).

## Figures and Tables

**Table 1:** Kinetic parameters of scFv-FGFR1 interaction measured with SPR. Sensor-immobilized FGFR1.D1-D2-D3-Fc was incubated with various concentrations of scFv-Fc proteins and kinetic parameters (ka, kd, and K_D_) were determined using BIAevaluation 4.1 software. * values from [16]. K_D_ errors represent standard deviation.

	scFvD2-Fc *	scFvK10-Fc	scFvL12-Fc
K_D_ (nmol/L)	0.59	9.41 ± 1.47	11.6 ± 1.25
k_on_ (M^−1^s^−1^)	6.47 × 10^5^	6.82 × 10^5^	4.08 × 10^5^
k_off_ (s^−1^)	3.84 × 10^−4^	6.42 × 10^−3^	5.45 × 10^−3^

## Supplementary Materials

Supplementary materials can be found at http://www.mdpi.com/1422-0067/19/5/1435/s1.

## Abbreviations

ADC	Antibody Drug Conjugates
FGFR1	Fibroblast Growth Factor Receptor 1
FGF	Fibroblast Growth Factor
EGF	Epidermal Growth Factor
