# Supplementary material for: High Affinity Promotes Internalization of Engineered Antibodies Targeting FGFR1
Source: Int J Mol Sci. 2018 May 10;19(5):1435. doi: 10.3390/ijms19051435 (PMC5983756; doi:10.3390/ijms19051435)
Supplement: Supplementary file 1 [file ijms-19-01435-s001.pdf]

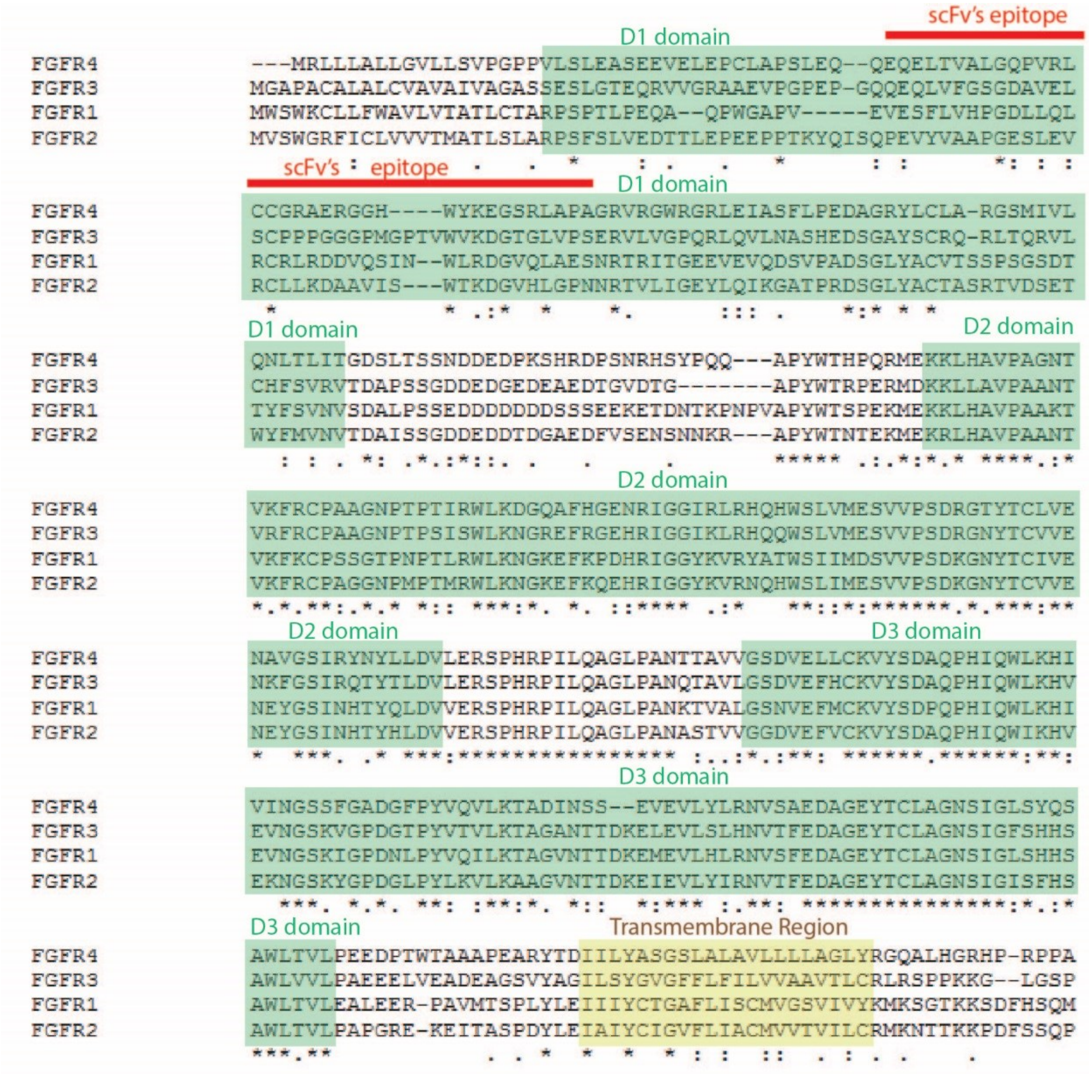

Figure S1. Multiple sequence alignment of the extracellular regions of FGF receptors (isoforms IIIc). Sequences of FGFR1 (NP_056934.2), FGFR2 (NP_000132.3), FGFR3 (NP_000133.1) and FGFR4 (NP_002002.3) were aligned with MUSCLE server (https://www.ebi.ac.uk/Tools/msa/muscle/). The boundaries of domains, transmembrane regions, and scFv's binding site were labeled on green, yellow and red, respectively.

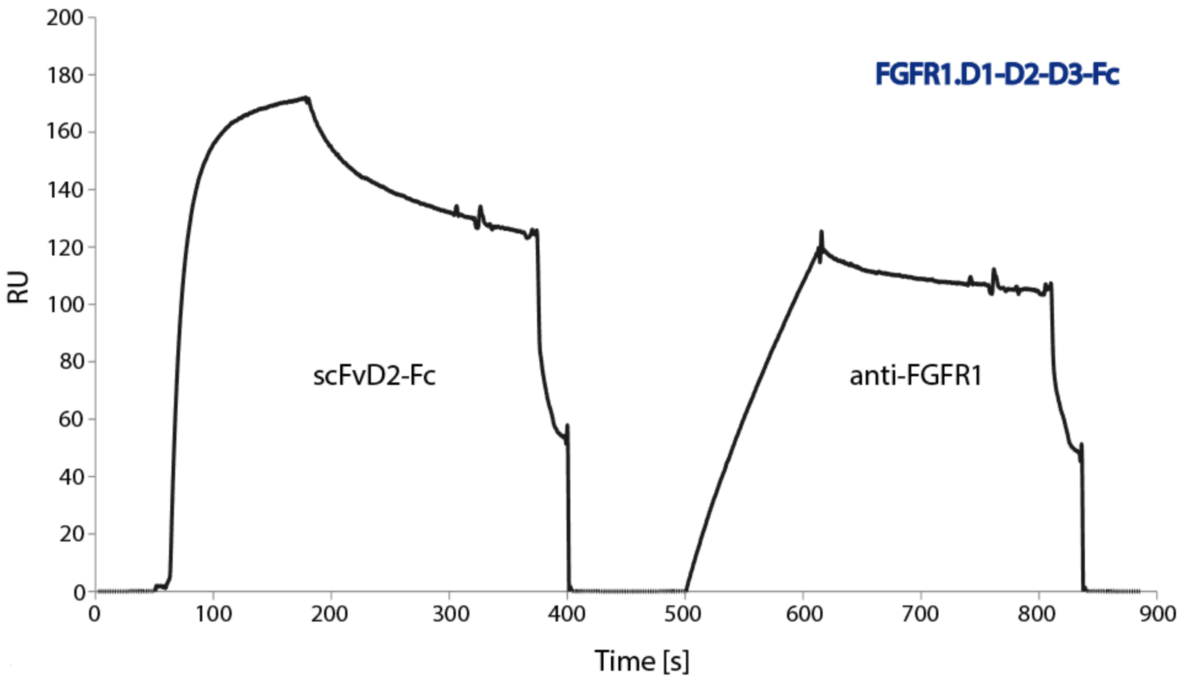

Figure S2. Specificity of scFv proteins towards FGFR1. Extracellular region of FGFR1 (FGFR1.D1-D2-D3-Fc) was immobilized on CM5 sensors and incubated with anti-FGFR1 antibody and scFvD2-Fc.

Supplementary Materials

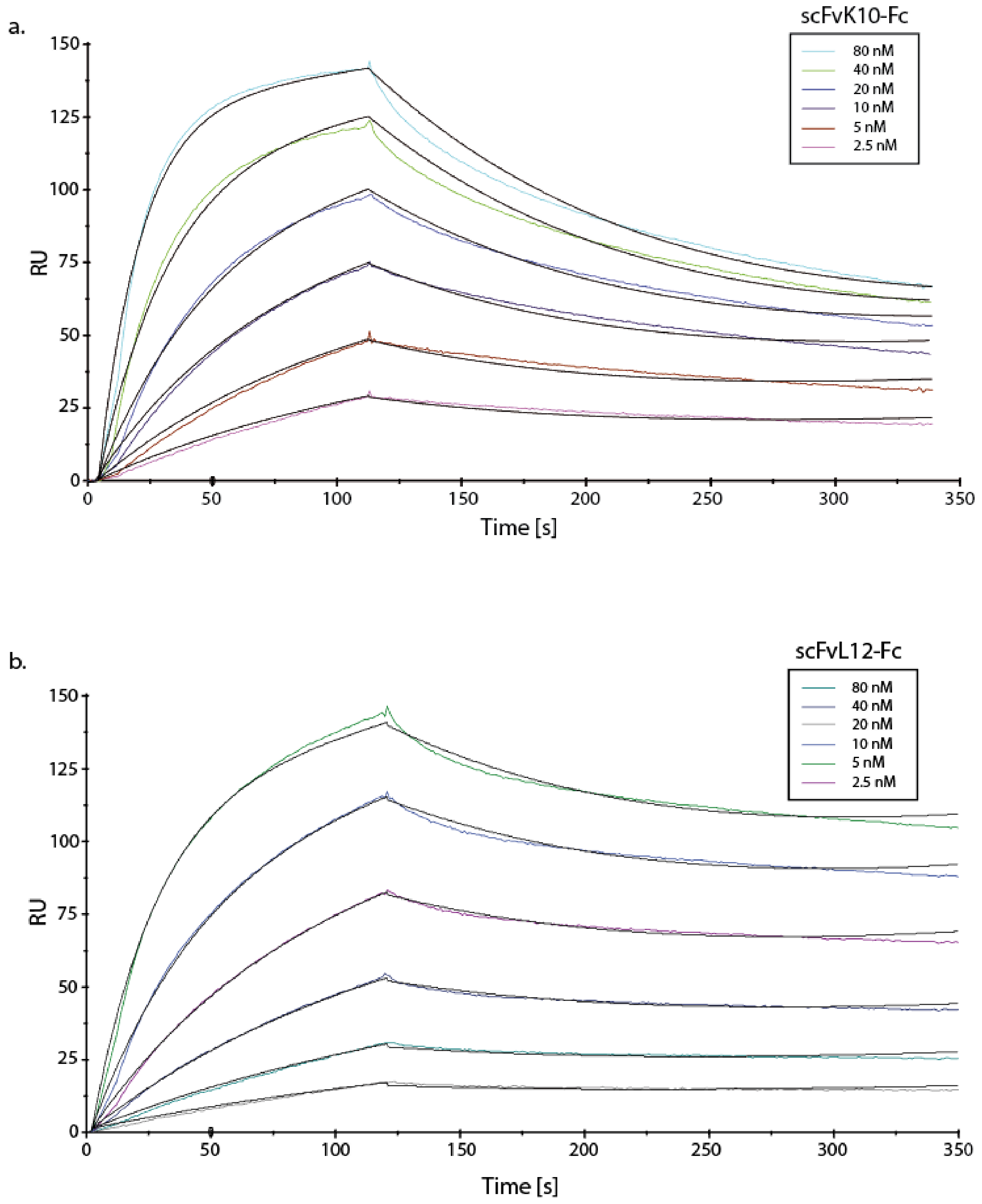

Figure S3. SPR sensograms with fitting curves (black lines) according to 1:1 Langmuir binding model with drifting baseline. Graphs were prepared using BIAevaluation 4.1 software.
